# Cold storage as part of a Varroa management strategy: effects on honey bee colony performance, mite levels and stress biomarkers

**DOI:** 10.1038/s41598-023-39095-5

**Published:** 2023-07-22

**Authors:** William G. Meikle, Vanessa Corby-Harris, Vincent Ricigliano, Lucy Snyder, Milagra Weiss

**Affiliations:** 1 Carl Hayden Bee Research Center, USDA-ARS, Tucson, AZ 85719 USA; 2 Honey Bee Breeding, Genetics, and Physiology Research, USDA-ARS, Baton Rouge, LA 70820 USA

**Keywords:** Agroecology, Ecosystem services

## Abstract

Placing honey bee colonies in cold storage has been proposed as a way to induce a pause in brood production as part of a Varroa mite treatment plan. Here, we exposed colonies to combinations of with or without an October cold storage period and with or without a subsequent miticide application. We then measured the effects of those treatments on colony-level variables (i.e. colony size, Varroa infestation level, survivorship and hive weight and temperature) and pooled individual-level variables that are associated with nutritional and stress responses. Colonies were assessed before and after cold storage, and again post winter, for a total duration of about 5 months, and the experiment was repeated. Brood levels were significantly lower after cold storage, and hive temperatures indicated that most or all brood had emerged after about two weeks in cold storage. However, Varroa levels at the end of the experiments in February were not significantly different among treatment groups. Colonies kept outside (not subjected to cold storage) and treated with a miticide had higher survivorship on average than any other treatment group, but no other group comparisons were significant, and long-term impact of cold storage on adult bee populations and on colony thermoregulation was low. The bee forage environment was also very different between the 2 years of the study, as rainfall and bee forage availability were much higher the second year. Colonies were over 2.5 times larger on average the second year compared to the first, both in terms of adult bee mass and brood area, and expression levels of nutrition and stress response genes were also significantly higher the second year. The results indicate that limited cold storage would likely have little long-term impact on most colony and individual measures of health, but for such a strategy to succeed levels of stressors, such as Varroa, may also need to be low.

## Introduction

The impact of *Varroa destructor* (hereafter “Varroa”) on colony health is well known^1,2^, and reducing Varroa infestations is a primary goal and major expense for beekeepers^3^. Managing Varroa in warmer areas in the western US is particularly challenging because colonies forage and produce more brood during winter months, and thus can facilitate Varroa population growth over a longer period, than those in other regions^4^. Varroa mites in brood cells are largely protected from many miticides, so the more brood there is during application, the larger the protected population of mites.

One way to reduce mite loads in honey bee colonies is to combine a miticide with brood reduction or “brood break”^5–7^. One way to do this is by caging the queen within the hive for at least 20 days^5–8^. As the existing brood hatches, the mites are forced out of the cells and are thus more susceptible to miticides^5,6^. While this can be accomplished in smaller apiaries, the labor that caging queens entails is difficult to implement on a commercial scale. Another way to induce a brood break is to place the colonies into cold storage conditions for extended periods^9^. The cold (< 8 °C) and dark conditions provide cues to the queen to cease oviposition^10^. As a result, brood in the hive emerges without replacement, and most mites in the hive are on the adult bees, fully exposed to miticides^5^.

Placing bee colonies in cold storage is a practice of long standing in the U.S., having first been mentioned in the early 1900s^9^. Commercial bee colonies are often placed in refrigerated warehouses during the winter to protect the colonies from weather events and to reduce the need for field management^10^. Colonies are typically placed inside dedicated warehouses in the late fall and removed just prior to shipping for almond pollination in late January or early February^11^.

It is unclear how the move from ambient conditions to cold storage affects the bees themselves, particularly in the southwestern U.S., where ambient temperature in the fall may exceed 30 °C. Colonies in ambient conditions in temperate climates respond to the gradual onset of winter by entering a distinct “overwintering” state, in which the queen ceases egg production and workers undergo sharp physiological changes^10^. One way to measure this shift is by measuring the expression levels of recently identified vitellogenin-like A genes, which have been shown consistent with elevated levels of vitellogenin, and are effective at early detection of the physiological shift to the overwintering state^12^. When colonies are placed abruptly into cold conditions, this gradual and innate overwintering process may be altered, impacting queen, adult worker bee, and colony health. Low temperatures can have a negative impact on worker development and learning^13,14^. If queen oviposition does not cease and worker thermoregulation is inadequate, the developing brood may suffer, thereby compromising foraging behavior of colonies placed in cold storage. Worker bees under cold storage conditions may undergo a high degree of stress, similar to that experienced under migratory management conditions^15^. The accumulation of oxidative damage to macromolecules is a known stress marker and can be measured via lipid peroxidation^15^ and protein carbonylation levels^16^. Cold stress has been shown to modulate the expression of genes involved in stress responses (*vitellogenin*, *superoxide dismutase*, *heat shock protein 70* and *heat shock protein 90*)^17^. However, honey bees in general are remarkably adept at dealing with sudden changes in the environment. Colonies can be placed in cold storage, removed 8 weeks later, shipped to a new location entirely and immediately produce brood, all while maintaining an adequate temperature regime^11^. While exposure to cold storage in the southwestern US in early October is likely to have some negative impacts on the bee population, colonies might quickly recover post-storage with fall forage or feeding. If so, colonies may benefit more from the improved Varroa control than what they lose from the stress of the cold storage.

**Figure 1:**
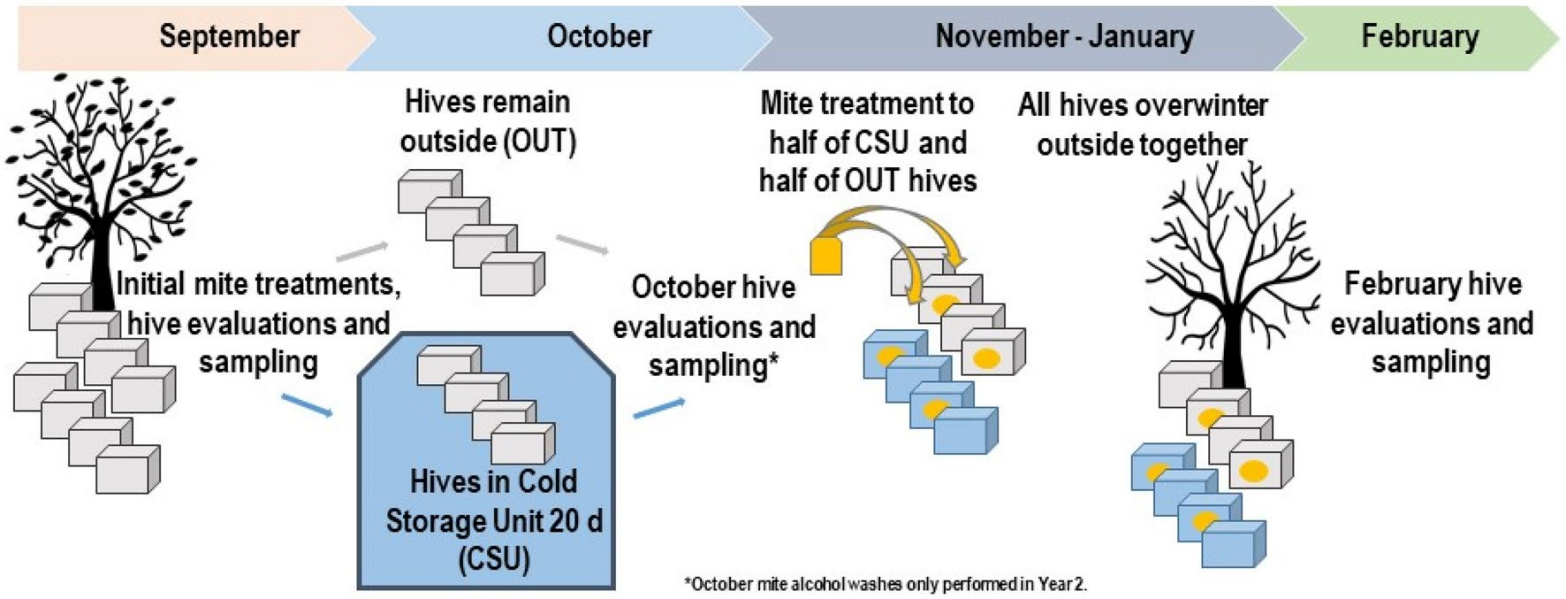
Schematic of general experimental design.

Our objective was to compare the Varroa infestation levels and long-term health status of honey bee colonies kept in ambient outdoor conditions (15/32 °C average nighttime/daytime temperatures) to colonies that were subjected to 3 weeks of dark cold storage conditions (constant 5 °C), both with and without a subsequent miticide treatment. We measured the effect of the treatment on mite levels over time, as well as the nutritional and stress status of individual bees before and after cold storage, and colony thermoregulation and growth after treatment (hereafter “treatment” refers to the combination of ± cold storage and ± post-storage miticide applied to each group). For an overview of the experimental design, please see Fig. 1.

## Results

### Colony level effects

**Figure 2:**
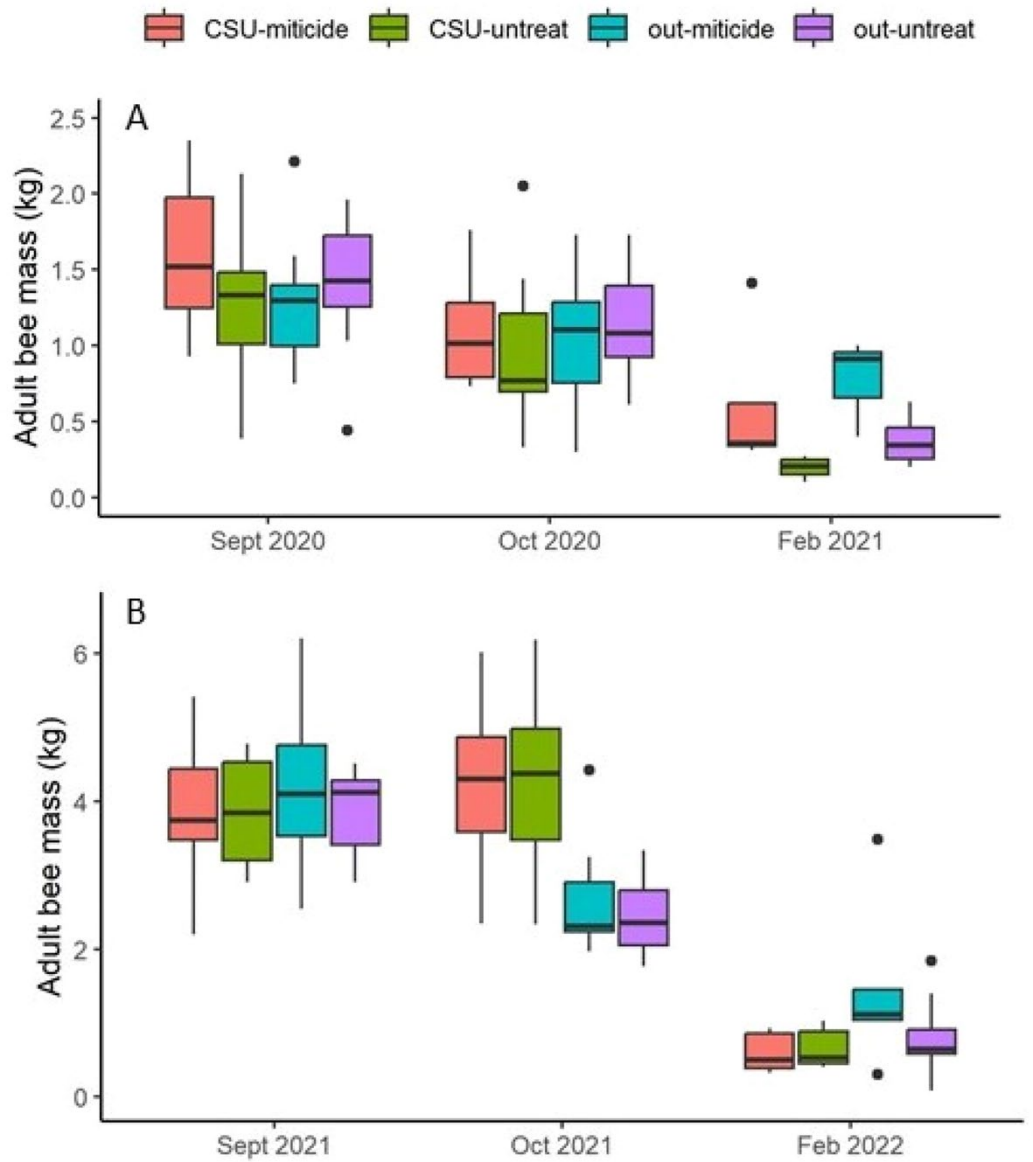
Adult bee mass in kg across the three sampling occasions. (**A**) 2020–21 experiment; (**B**) 2021–22 experiment. Note that the scales of the Y axes are different: 0–2.5 kg in 2020–21 and 0–6.5 kg in 2021–22. Boxes are defined as 1.58 × IQR/n^0.5^, where IQR is the inter-quartile range and n is the number of data. Points represent data considered outliers within the respective treatment group. “CSU” indicates cold storage, “out” indicates colonies that were kept out of cold storage, “miticide” indicates colonies received miticide application just after cold storage, and “untreat” indicates colonies that did not receive miticide application. Only one treatment comparison, “CSU-miticide” ×vs “out-untreat” was significant.

**Figure 3:**
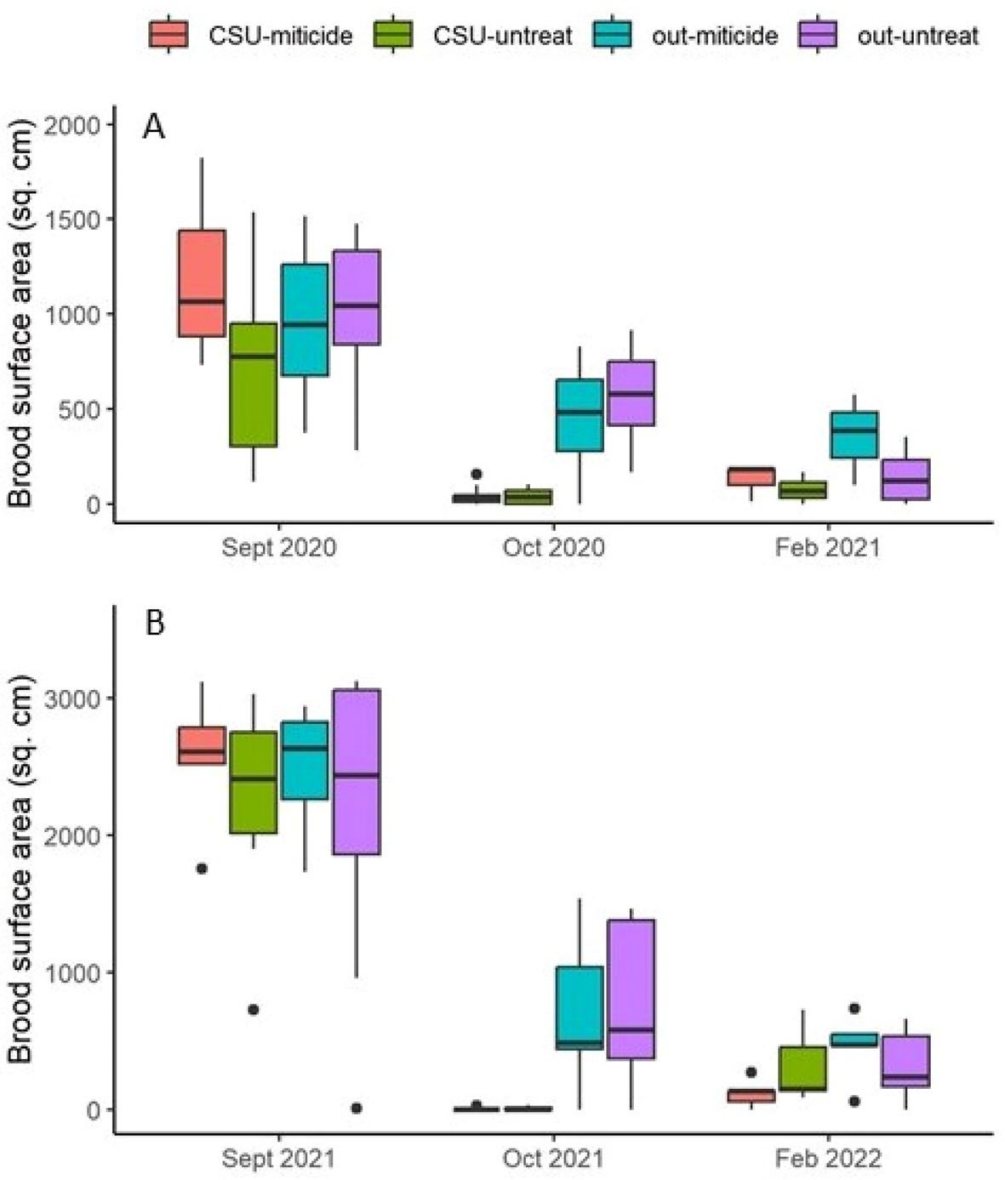
Brood surface area in cm^2^ across the three sampling occasions. (**A**) 2020–21 experiment; (**B**) 2021–22 experiment. Note that the scales of the Y axes are different: 0–2000 sq. cm in 2020–21 and 0–3500 sq. cm in 2021–22. Boxes are defined as 1.58 × IQR/n^0.5^, where IQR is the inter-quartile range and n is the number of data. Points represent data considered outliers within the respective treatment group. “CSU” indicates cold storage, “out” indicates colonies that were kept out of cold storage, “miticide” indicates colonies received miticide application just after cold storage, and “untreat” indicates colonies that did not receive miticide application. All pairwise comparisons were significant except one: “out-miticide” vs “out-untreat.”

In September prior to cold storage, colonies in 2020 had 1.38 ± 0.07 kg of adult bees and 961 cm^2^ sealed brood on average while colonies in 2021 had significantly more, with 3.95 ± 0.14 kg adult bees and 2410 cm^2^ sealed brood (adult bees: t = 13.12, d.f. = 82, P < 0.0001; sealed brood: t = 10.05; d.f. = 82; P < 0.0001) (Figs. 2 and 3). Colonies in 2020 were smaller due in a large part to the low rainfall that year.

In the initial ANOVA analysis, involving data only from Sept. and Oct. and thus only addressing the effects of cold storage, cold storage had a significant effect on both adult bee mass (P = 0.0052; effect size: 0.506 ± 0.494) and brood area (P < 0.0001; effect size: 2.718 ± 1.954). While the year of the experiment explained a significant amount of variance in adult bee mass (P = 0.0010; effect size: 0.751 ± 0.680) it did not in the case of brood area (P = 0.63) after controlling for pre-existing differences. In 2020 colonies put in cold storage lost 29% of their mass of adult bees between the September and October sampling occasions, compared to an 18% loss during that same period of time for colonies kept outside. In 2021 colonies put in cold storage actually increased adult bee mass by about 9% while colonies kept outside lost 36%. By February in both years colonies had lost an average of 80% of their adult bee mass if they had been in cold storage and 60% otherwise.

**Figure 4:**
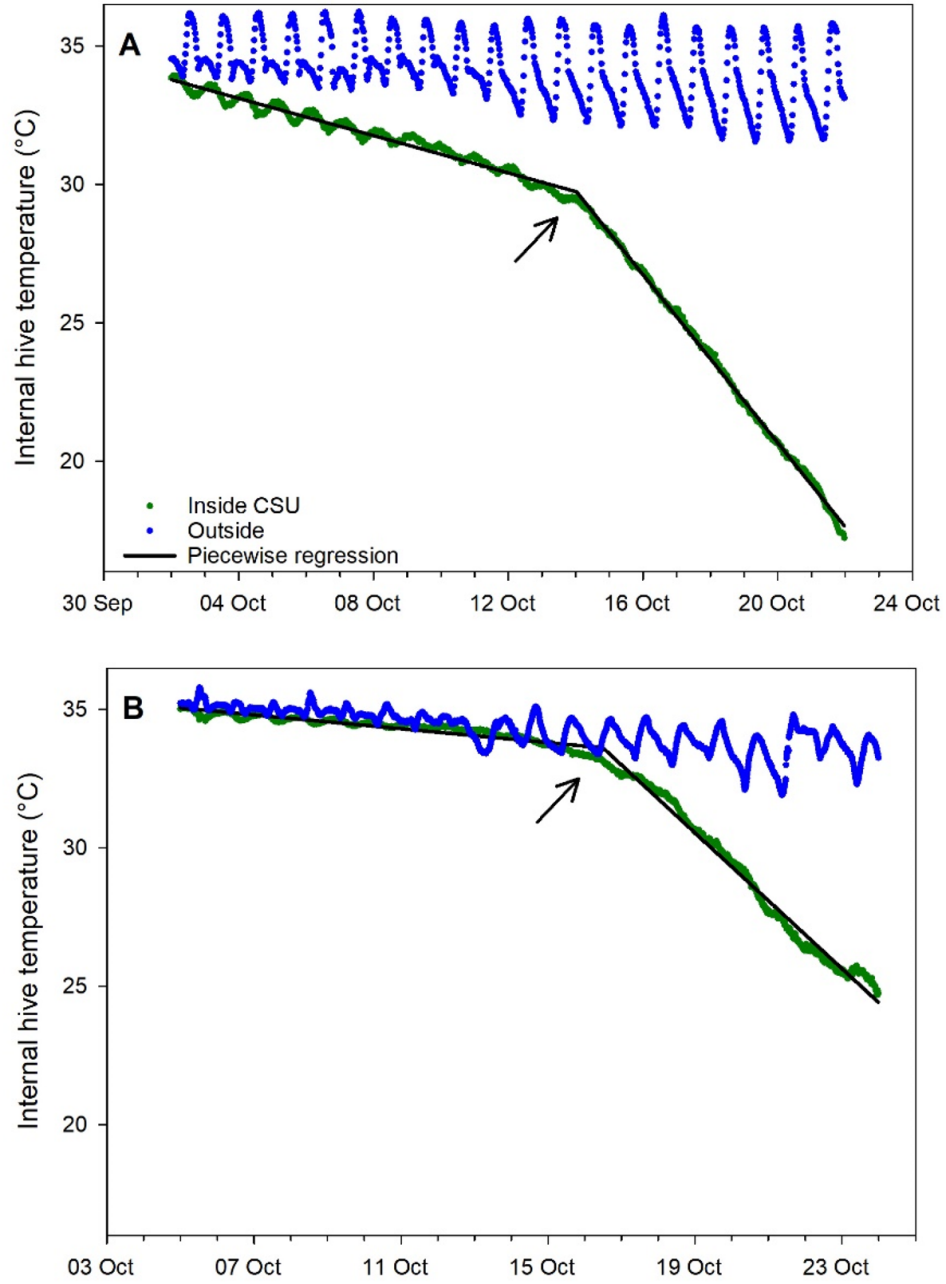
Average internal hive temperature with a fit piecewise regression. Shown are average raw data from hives inside the CSU and outside (ambient conditions) with a regression fit to the CSU data. (**A**) 2020–21 experiment (30 min. data intervals); (**B**) 2021–22 experiment (15 min. data intervals). Arrows show break point (estimated time at which sealed brood had emerged).

Piecewise regression with a single break point of the hive temperature data during cold storage fit the data well (average ± s.e. r^2^ = 0.93 ± 0.03 and 0.96 ± 0.01 for 2020 and 2021 respectively) (Fig. 4). Break points showed that average temperature abruptly decreased after an average of 12.9 ± 0.8 days in cold storage in 2020 and 12.5 ± 0.7 days in 2021.

**Figure 5:**
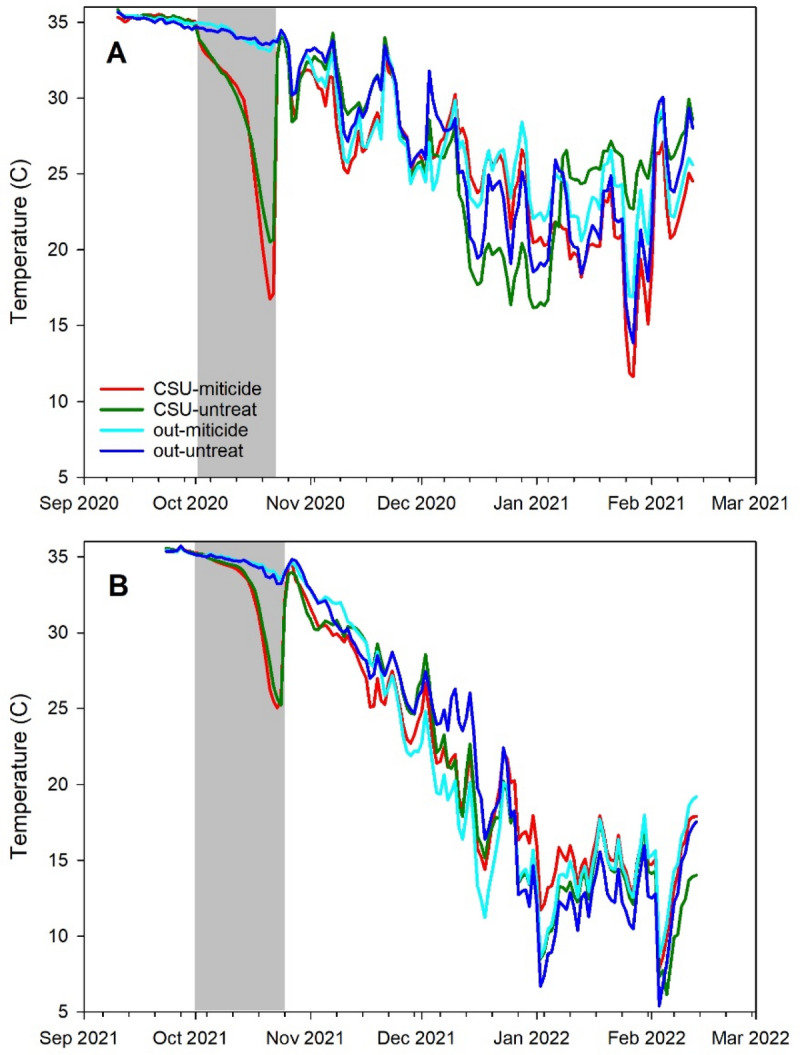
Average internal hive temperature. (**A**) 2020–21 experiment; (**B**) 2021–22 experiment. “CSU” indicates cold storage, “out” indicates colonies that were kept out of cold storage, “miticide” indicates colonies received miticide application just after cold storage, and “untreat” indicates colonies that did not receive miticide application. The gray bar indicates the cold storage period. Only one treatment comparison, “CSU-miticide” vs “out-untreat” was significant.

The MANOVA analyses, involving data from Sept. through Feb. and involving ± cold storage and  ± miticide, showed that treatment had a significant effect on adult bee mass, brood area, and average daily hive temperature (Table 1). Neither treatment nor year affected changes in temperature amplitudes (P = 0.81 and P = 0.17, respectively). Post hoc analysis showed that treatment effects for adult bee mass and average daily hive temperature were limited to a single comparison: colonies subjected to both cold storage and miticide had significantly lower adult population sizes yet higher average temperatures than those subjected to neither (Table 2). In contrast, post hoc analysis of log brood areas showed many significant pairwise comparisons with substantial effect sizes. Colonies in 2021 had significantly more brood (effect size 2.192 ± 1.567) but lower temperatures (effect size 0.121 ± 0.114) on average than colonies in 2020 (Fig. 5).

**Table 1:** MANOVA results for the factors treatment group (± cold storage and ± miticide), year of experiment (2020 and 2021), sampling occasion (Oct. and Feb.) and two-way interactions for colony level response variables. Response variables and covariates were log transformed. “Pre adult mass” and “Pre brood” indicate values measured prior to treatment and were used as covariates to control for pre-existing differences. Effects with significant P values are in bold.

Response variable	Effect	Num DF	Den DF	F value	Pr > F
Log adult bee mass	**Treatment group**	**3**	**78.5**	**3.73**	**0.0146**
	Year	1	83.8	3.08	0.0829
	**Sampling occ**	**1**	**71.6**	**228.02**	**< 0.0001**
	**Treat × Year**	**3**	**76.2**	**3.56**	**0.0182**
	**Treat × Sampling occ**	**3**	**69.8**	**8.82**	**< 0.0001**
	**Year × Sampling occ**	**1**	**68.5**	**11.83**	**0.0010**
	**Pre adult mass**	**1**	**82.0**	**15.05**	**0.0002**
Log brood surface area	**Treatment group**	**3**	**54.8**	**35.29**	**< 0.0001**
	**Year**	**1**	**70.9**	**4.89**	**0.0302**
	Sampling occ	1	71.3	1.00	0.3207
	Treat × Year	3	54.0	0.03	0.9944
	**Treat × Sampling occ**	**3**	**69.8**	**9.16**	**< 0.0001**
	Year × Sampling occ	1	67.4	3.16	0.0798
	Pre brood	1	84.5	0.43	0.5161
Log daily temperature	**Treatment group**	**3**	**235.8**	**3.45**	**0.0173**
	**Year**	**1**	**322.0**	**9.37**	**0.0024**
	**Day**	**30**	**1886**	**60.42**	**< 0.0001**
	**Treat × Year**	**3**	**228.2**	**3.26**	**0.0224**
	**Treat × Day**	90	1885	1.12	0.2035
	**Year × Day**	**30**	**1887**	**60.84**	**< 0.0001**
	**Pre adult mass**	**1**	**497.5**	**4.29**	**0.0389**
Average daily hive weight change	Treatment group	3	776.1	1.59	0.1909
	Year	1	788.1	3.67	0.0559
	**Day**	**52**	**3449**	**62.12**	**< 0.0001**
	**Treat × Year**	**3**	**793.8**	**4.98**	**0.0020**
	**Treat × Day**	**156**	**3471**	**1.87**	**< 0.0001**
	**Year × Day**	**52**	**3449**	**50.02**	**< 0.0001**
	**Pre adult mass**	1	787.8	0.20	0.6518

**Table 2:** Pairwise post hoc comparisons and effect size for colony level response variables in which there was a significant treatment strategy main effect. “Group 1” and “Group 2” refer to the two groups in the comparison. “Cold storage” column indicates whether or not group was placed in the cold storage facility; “Miticide” column indicates whether or not group was treated with miticide after the storage period at the end of October; “N1” and “N2” columns show sample sizes for Groups 1 and 2, respectively. Effect size (with 95% confidence intervals) were calculated using Hedge’s g: t × ((n1 + n2)/(n1 × n2))^0.5^ where t is the t value of the contrast. Contrasts which do not have zero in the effect size s.e. interval are in bold.

Response variable	Group 1		Group 2		N1	N2	t value	Effect size	Effect size s.e
	Cold storage	Miticide	Cold storage	Miticide					
Log adults	Yes	No	Yes	Yes	29	32	1.49	0.382	0.570
	Yes	No	No	No	29	29	− 1.61	0.423	0.595
	Yes	No	No	Yes	29	39	0.91	0.223	0.505
	**Yes**	**Yes**	**No**	**No**	**32**	**29**	− **3.15**	**0.808**	**0.759**
	Yes	Yes	No	Yes	32	39	− 0.69	0.165	0.481
	No	No	No	Yes	29	39	2.65	0.650	0.660
Log brood	**Yes**	**No**	**Yes**	**Yes**	**28**	**32**	− **1.24**	**0.321**	**0.283**
surface area	**Yes**	**No**	**No**	**No**	**28**	**29**	− **7.8**	**2.067**	**0.789**
	**Yes**	**No**	**No**	**Yes**	**28**	**39**	− **7.54**	**1.868**	**0.706**
	**Yes**	**Yes**	**No**	**No**	**32**	**29**	− **6.93**	**1.777**	**0.688**
	**Yes**	**Yes**	**No**	**Yes**	**32**	**39**	− **6.66**	**1.589**	**0.615**
	No	No	No	Yes	29	39	1.03	0.253	0.261
Log daily temperature	Yes	No	Yes	Yes	483	562	− 1.31	0.081	0.068
	Yes	No	No	No	483	476	1.87	0.121	0.077
	Yes	No	No	Yes	483	620	− 0.81	0.049	0.063
	**Yes**	**Yes**	**No**	**No**	**562**	**476**	**2.91**	**0.181**	**0.089**
	Yes	Yes	No	Yes	562	476	0.51	0.032	0.063
	No	No	No	Yes	476	476	− 2.65	0.172	0.089

**Figure 6:**
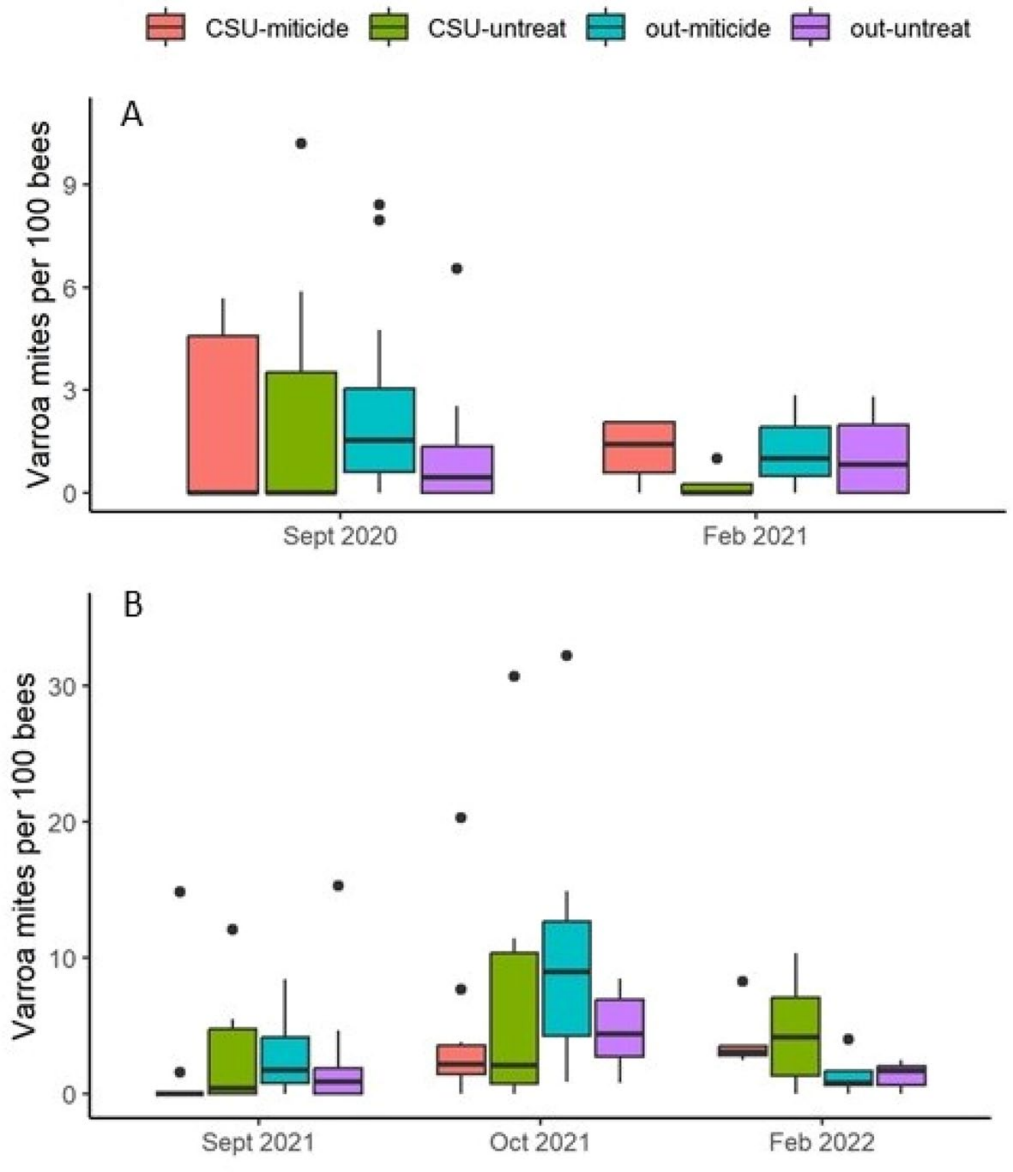
Varroa mites per 100 bees. (**A**) 2020–21 experiment; (**B**) 2021–22 experiment. Owing to the small size of the adult bee populations after cold storage, bee samples were not collected in October 2020. Note that the scales of the Y axes are different: 0–11 mites per 100 bees in 2020–21 and 0–35 mites per 100 bees in 2021–22. Boxes are defined as 1.58 × IQR/n^0.5^, where IQR is the inter-quartile range and n is the number of data. Points represent data considered outliers within the respective treatment group. “CSU” indicates cold storage, “out” indicates colonies that were kept out of cold storage, “miticide” indicates colonies received miticide application just after cold storage, and “untreat” indicates colonies that did not receive miticide application. No significant differences between treatment groups were observed.

Varroa levels were not normally distributed, either raw or transformed, and thus subjected to pairwise comparisons (α = 0.05) using a rank sum test. Pre-treatment Varroa infestation levels in September were not different between years, but post-treatment infestation levels across treatment groups in February were different between years (P = 0.011) with a median value of 0.80 and 2.31 mites per 100 bees for Feb. 2021 and Feb. 2022, respectively (Fig. 6). The change in Varroa infestation levels over time (subtracting February values from September values) were not significantly different between years (P = 0.168). Considering all treatment groups across years in an omnibus analysis and applying α = 0.05/6 = 0.0083 to each comparison, no comparisons among treatment groups were significant (P = 0.032–0.837). The lowest P value, 0.032, was obtained from the (+ cold storage − miticide) group vs. (− cold storage + miticide) group comparison, with median values of 2.49 and 0.84 mites per 100 bees, respectively.

**Figure 7:**
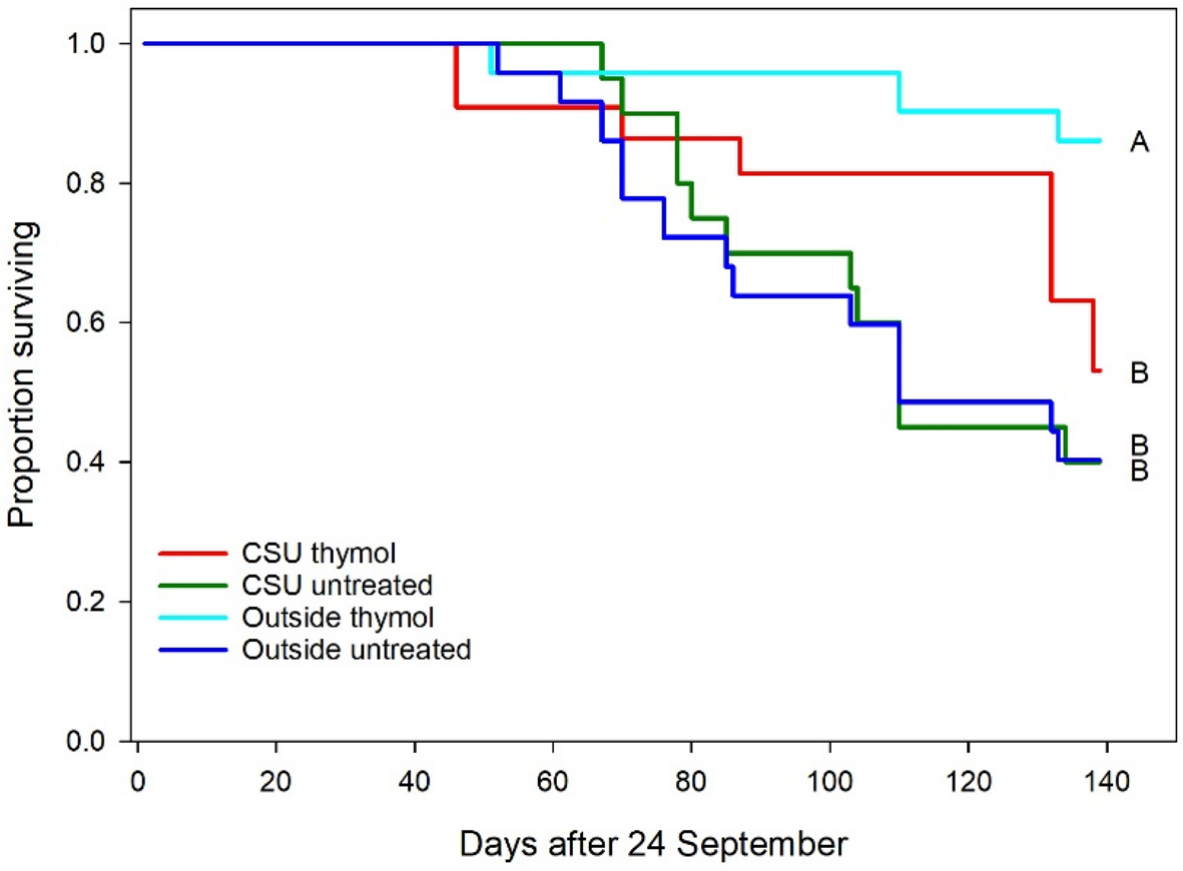
Average colony survivorship over the course of the two experiments (starting date was set to 24 September for both years) “CSU” indicates cold storage, “Outside” indicates colonies that were kept out of cold storage, “miticide” indicates colonies received thymol treatment just after cold storage, and “untreated” indicates colonies that did not receive thymol treatment. The proportional hazards of groups associated with lines followed by different letters were significant.

In 2020 21 of 50 of the colonies (42%) starting in September were still alive at the final evaluation the following February, whereas in 2021 24 of 40 (60%) of the colonies survived. The Cox regression analysis of colony survivorship did not show any significant differences with respect to year or the interaction of year and treatment, but within the treatment factor, colonies treated with thymol and kept outside had significantly higher survivorship than any other groups (Fig. 7). Considering colonies kept outside and treated with thymol as the reference group, the proportional hazard was 2.16 ± 0.81 (P = 0.008) compared to outside colonies left untreated, 1.70 ± 0.83 (P = 0.041) compared to colonies in cold storage and treated with thymol, and 1.78 ± 0.85 (P = 0.036) compared to colonies in cold storage and left untreated.

### Oxidative stress accumulation

**Figure 8:**
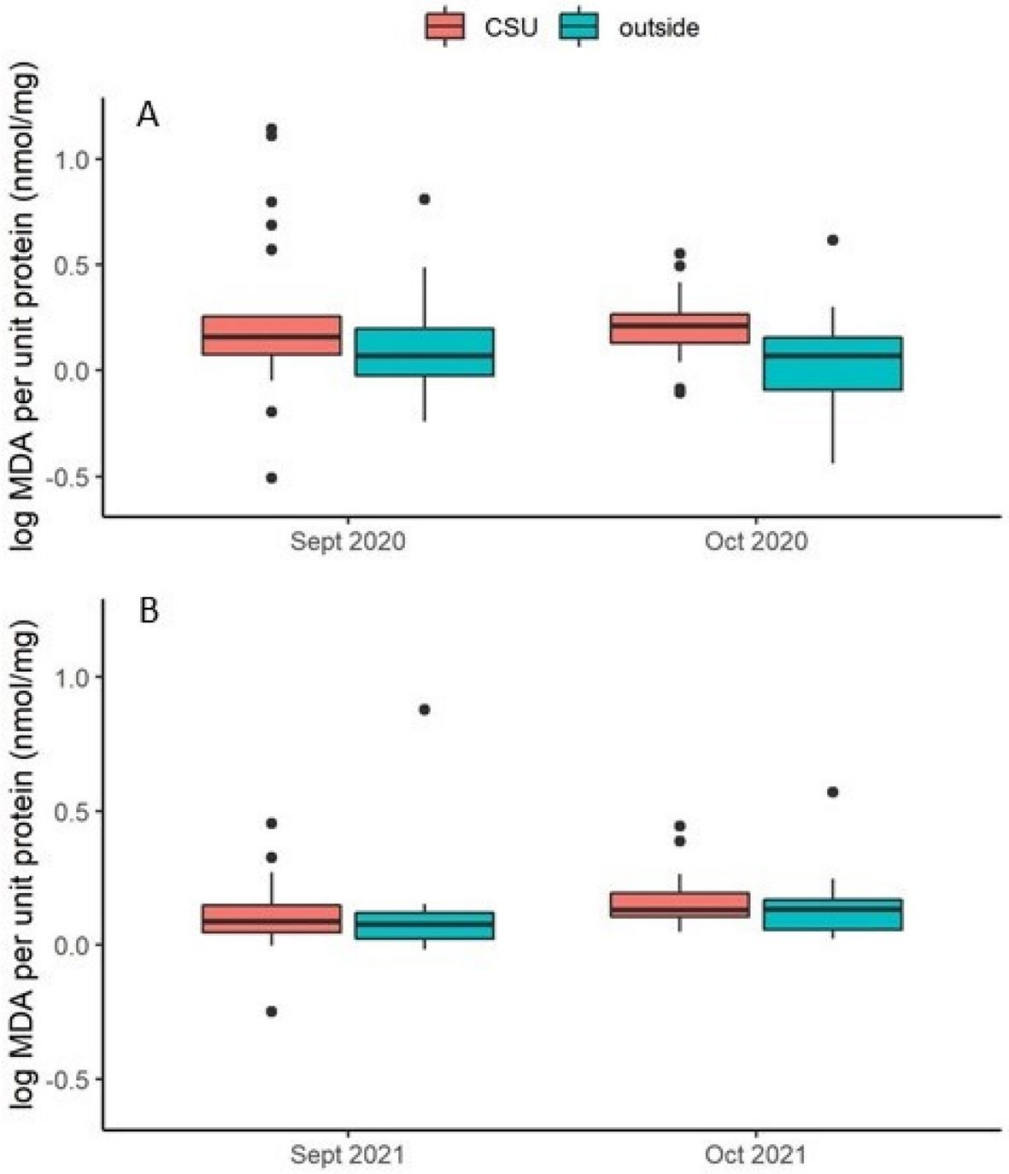
Log MDA per unit protein (nmol/mg) before and after cold storage. (**A**) 2020–21 experiment; (**B**) 2021–22 experiment. Boxes are defined as 1.58 × IQR/n^0.5^, where IQR is the inter-quartile range and n is the number of data. Points represent data considered outliers within the respective treatment group. “CSU” indicates colonies that were placed in the cold storage unit and “outside” indicates colonies that remained outdoors. Small significant differences were observed between the treatment groups “CSU” and “outside.”

Log-transformed MDA values showed a weak effect of cold storage and no effect of year (Tables 3 and 4, Fig. 8). Log-transformed carbonyl levels were not affected by cold storage but the effect size of year was strong, indicating that colony size and/or condition played a far larger role in carbonyl levels than did exposure to cold storage as it was measured here.

**Table 3:** F ratios and P values for cold storage (+ or −), year (2020 or 2021), and their interactions in ANOVAs for individual level response variables. Response variables and covariates had been transformed as log values. “Cold storage” indicates exposure to cold storage; “Pre-treat values” indicates the values obtained from sample prior to cold storage in September and were used as covariates; “VG” indicates vitellogenin; “Sup. dismut.” indicates superoxide dismutase; “HSP” indicates heat shock protein. Effects with significant P values are in bold.

Response variable	Cold storage		Year		Cold storage × Year		Pre-treat values	
	F	P	F	P	F	P	F	P
MDA	**5.77**	**0.0187**	0.96	0.3304	3.17	0.0788	0.36	0.5484
Carbonyl	2.92	0.0917	**11.95**	**0.0009**	2.99	0.0879	0.62	0.4337
Vitellogenin	0.57	0.4509	**22.63**	**< 0.0001**	0.03	0.8554	0.00	0.9827
VG-like A	**4.81**	**0.0314**	**38.34**	**< 0.0001**	0.05	0.8251	1.79	0.1856
Catalase	2.05	0.1564	**55.61**	**< 0.0001**	0.67	0.4141	0.82	0.3688
Sup. dismut	0.02	0.8866	**48.12**	**< 0.0001**	1.71	0.1951	0.17	0.6847
HSP 70	1.15	0.2872	**7.94**	**0.0073**	**5.91**	**0.0174**	0.31	0.5816
HSP 90	1.28	0.2623	**25.32**	**< 0.0001**	0.62	0.4324	0.04	0.8455

**Table 4:** Pairwise post hoc comparisons and effect size for individual level response variables in which there was a significant effect of cold storage and/or year of study. “Group 1” and “Group 2” refer to the two groups in the comparison. “CSU” indicates group placed in cold storage unit; “outside” indicates group placed in ambient conditions outside the CSU; “N1” and “N2” columns show sample sizes for Groups 1 and 2, respectively. Effect size (with 95% confidence intervals) were calculated using Hedge’s g: t × ((n1 + n2)/(n1 × n2))^0.5^ where t is the t value of the contrast. Contrasts which do not have zero in the effect size s.e. interval are in bold.

Fixed effect	Response variable	Group 1	Group 2	N1	N2	t value	Effect size	Effect size s.e
Cold treat	MDA	CSU	Outside	42	42	2.40	0.524	0.564
	VG-like A	CSU	Outside	42	40	− 2.19	0.484	0.550
Year	**Carbonyl**	**2020**	**2021**	**46**	**38**	− **3.46**	**0.758**	**0.682**
	**Catalase**	**2020**	**2021**	**45**	**37**	− **7.46**	**1.656**	**1.235**
	**Sup. dismut**	**2020**	**2021**	**45**	**37**	− **6.94**	**1.540**	**1.160**
	**HSP 70**	**2020**	**2021**	**45**	**37**	− **2.82**	**0.626**	**0.617**
	**HSP 90**	**2020**	**2021**	**45**	**37**	− **5.03**	**1.116**	**0.893**
	**Vitellogenin**	**2020**	**2021**	**45**	**37**	− **4.76**	**1.056**	**0.856**
	**VG-like A**	**2020**	**2021**	**45**	**37**	− **6.19**	**1.374**	**1.053**

### Gene expression

**Figure 9:**
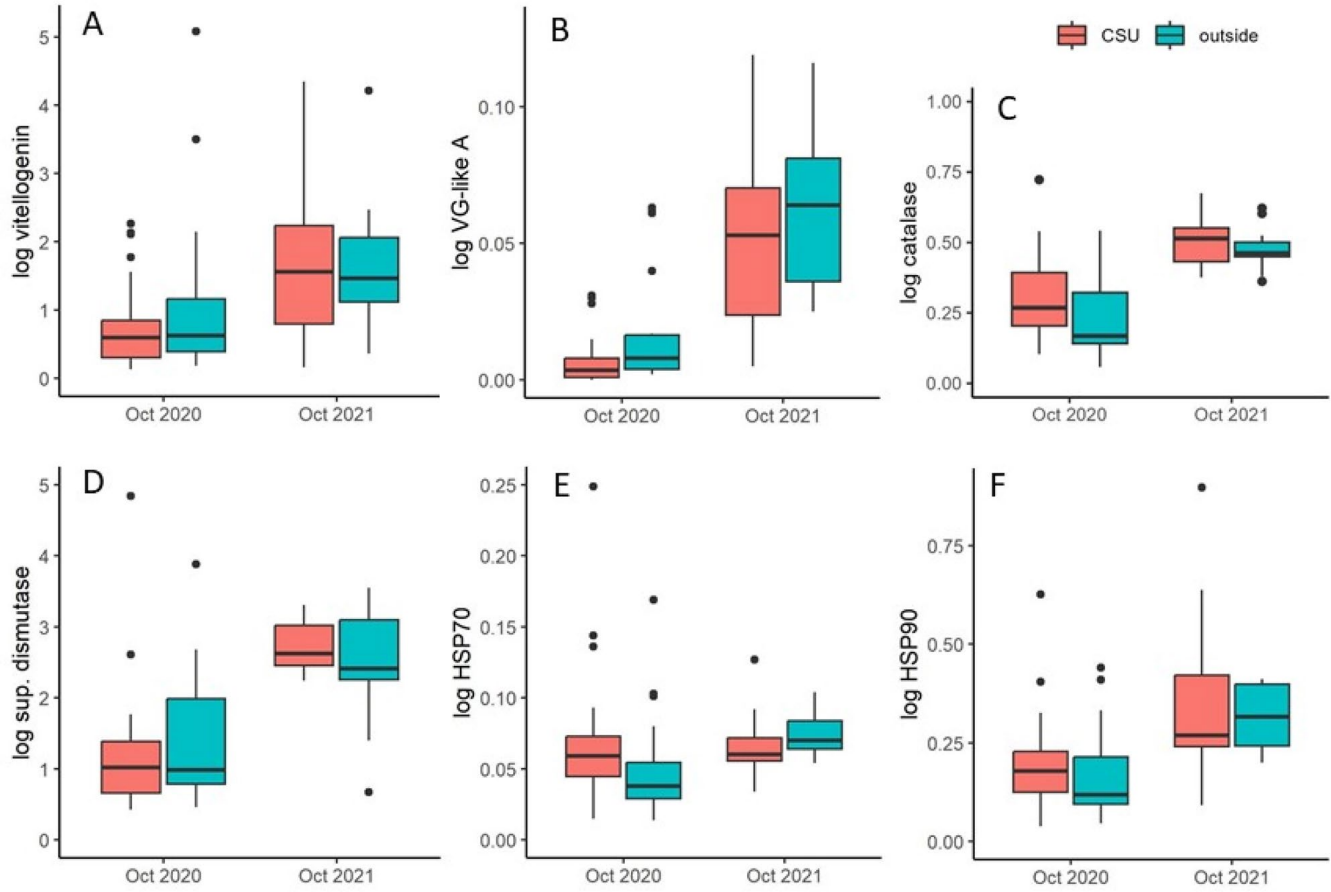
Relative gene expression after the cold storage period for colonies placed in the cold storage unit (“CSU”) and those kept outside the cold storage unit in ambient conditions (“out”) for both years of the study. (**A**) vitellogenin; (**B**) vitellogenin-like-A; (**C**) catalase; (**D**) superoxide dismutase; (**E**) heat shock protein 70; and (**F**) heat shock protein 90. “CSU” indicates colonies that were placed in the cold storage unit and “out” indicates colonies that remained outdoors. All comparisons between “Oct 2020” and “Oct 2021” were significant with a significant effect size.

Of the six genes measured in the study, only vitellogenin-like-A expression was significantly affected by cold storage, and that effect was found to be weak. However, the expression of all six genes was strongly affected by the year of the study (Fig. 9). As noted above in the colony level results, colonies had significantly larger adult bee populations and more sealed brood in 2021 than in 2020, suggesting the colonies were healthier in 2021. Post hoc pairwise comparisons showed that the effect size of year was significant for all gene expression examined in the study.

### Deformed wing virus levels

Levels were high for both DWV A and DWV B in the second year prior to cold storage, averaging 7.44 ± 0.29 and 8.08 ± 0.26 log genome equivalents/100 ng of RNA, respectively. Values were somewhat higher after cold storage (8.49 ± 0.29 and 9.09 ± 0.26 log genome equivalents/100 ng of RNA, respectively) but cold storage was not a significant factor (P = 0.96 for DWV-A and P = 0.12 for DWV-B).

## Discussion

The main objectives of this research were to evaluate a Varroa treatment strategy in terms of efficacy and its impact on honey bee colony health. The strategy involved exposing colonies to cold storage in the fall for a short period (no longer than 21 days) followed by a miticide application. The rationale was that cold storage would induce the colonies to stop brood production and once the remaining sealed brood emerged, most or all of the Varroa in the colony will either be in open frame cells or attached to adult bees^18^ and thus susceptible to the miticide. The strategy combines honey bee colony management methods already in common use by many commercial beekeepers.

The combined treatment strategy in this case was not effective at reducing Varroa infestations. The change in Varroa infestation levels between September, prior to the start of the experiments, and the following February, were not significantly affected by treatment. Beekeepers tend to pay more attention to the absolute Varroa levels rather than the changes in Varroa levels, and the absolute February Varroa levels were not significantly different among treatment groups (sample sizes at that point were small owing to colony mortality).

Cold storage did succeed in greatly reducing or eliminating sealed brood, as evidenced by the very low brood levels among those colonies post storage. Internal hive temperature is also a reliable indicator of brood production^19,20^ and abrupt decreases were detected, using piecewise regression, 13–14 days into cold storage, likely indicating the final emergence date of the sealed brood observed before cold storage. The sharply decreasing temperatures after that estimated final brood emergence date would correspond to a similarly decreasing volume under strict temperature control by the cluster^19^, which no longer had brood to stimulate strong thermoregulation. Other colony-level effects were few: significant treatment effects on log adult bee mass and log average daily temperature were limited to a single comparison in which colonies subjected to both cold storage and miticide had lower adult bee masses and higher average temperatures than colonies subjected to neither. Survivorship analysis showed that colonies kept outside and then treated with thymol had significantly higher survivorship than colonies in any other treatment group, suggesting that cold storage as implemented here did not have a positive effect at the colony level.

The lack of treatment effectiveness against Varroa may have one or more explanations. One possibility is that local environmental conditions at the apiaries at the end of the cold storage period, i.e. dry with essentially no forage, were not conducive to colony recovery. However, this is not likely since all colonies had sufficient honey reserves and consumed protein patty immediately after cold storage. A second possibility is that the Varroa levels prior to storage were too high for successful treatment in this manner, despite miticide application prior to storage. Comparatively high Varroa infestation levels are common in the southwestern US^4^. In this case 26% of the colonies in the first year and 29% of the colonies in the second year had Varroa infestation levels in excess of 3 mites per 100 bees, which has been considered a threshold for late summer/early fall application^21^. Some colonies in both years were well in excess of that threshold. Varroa mites cause many health problems in honey bee colonies and one of the most important is the transmission to adult bees of Deformed Wing Virus^22^. DWV levels were high prior to cold storage in the second year of the study (they were not measured the first year), and cold storage treatment itself did not have a significant effect. The high Varroa infestation levels combined with high levels of Varroa-transmitted viruses suggest that this treatment strategy was not ideal for those colonies.

A third possibility is that some parameters, such as the timing (in this case starting early October) and duration (in this case 20–22 days), were not optimal and need to be changed for the strategy to be effective. Placing colonies in cold storage earlier, such as mid September, would mean the environment post cold storage would be somewhat different, and shortening the storage period, even by just a few days, may reduce stress. In this case temperature data indicated that most brood had emerged after about two weeks in cold storage. Applying the cold storage earlier in the year would also allow an earlier application of miticide post cold storage, which may also increase chances for success.

While the strategy as tested here was not successful in reducing February mite levels, some information could be gleaned on the effects of stressors on colony and individual health. Regarding colony level data from the start of the experiments until the following February, adult bee mass, sealed brood area and average daily within-hive temperature were all significantly affected by the treatment strategy. That so few significant differences were observed among treatment groups suggests that colony-level health may be overall robust with respect to stress caused by cold storage and miticide application. Brood area was more sensitive to the treatment, which was not surprising given the explicit objective of the cold storage was to temporarily reduce brood production, and indeed colonies that were placed in cold storage had significantly lower brood levels, even as late as February, than colonies that did not. Adult bee masses and brood areas were significantly different between years of the study; the ANOVA analyses showed no significant effect of year but in those cases pre-treatment values were used as covariates to control for initial conditions and much of the variance associated with year could be attributed to those initial conditions.

Cold storage itself had a limited impact on adult worker bee health status but differences between the two years of the study were substantial. In general, colony-level gene expression was strongly affected by season and the nutritional landscape. In a previous study, a pooled-sampling approach indicated that large, healthy colonies exhibit higher gene expression levels of *vg, vg-like-A, superoxide dismutase, catalase, and glutathione-s-transferase1*^23^. In this study, only *vg-like-A* expression showed a significant effect of cold storage and the effect size was found to be small. Bees from colonies exposed to cold storage had significantly higher MDA levels, indicating higher levels of oxidative stress, although the effect was also small.

In contrast, the effects of year were significant, and large, for all gene expression response variables as it was with colony-level variables. Expression of genes for heat shock proteins and antioxidant enzymes were significantly higher in 2021 than in 2020, suggesting healthier colonies in 2021, but those bees also had significantly higher carbonyl levels with a large effect size, indicating expression of oxidative stress. Colony-level measures of adult bee mass and brood area were considerably higher in 2021 than in 2020, indicating that colonies were more robust, but Varroa levels were also higher.

The greater expression of vitellogenin and vg-like-A lipoproteins in 2021 would be consistent with a more diutinus state than bees in 2020, irrespective of the cold storage exposure^12^ but there was little additional evidence of a diutinus state; colonies in September in 2021 had about 2.9 times as many adult bees and about 2.5 times as much brood as those in 2020 so relative brood production was not much lower, as would be expected. A simpler explanation may be that colonies had better nutrition in 2021 than 2020, even if stress levels were higher. In 2020, summer rainfall (May to September) was about 42 mm in the Tucson area, whereas in 2021 it was about 325 mm^24^. This difference in rainfall resulted in a marked difference in forage availability, both in terms of quantity and duration. The increased rainfall and resulting forage levels in 2021 resulted in much larger colonies and support the conclusion of a better nutritional status among adult bees that year. The large differences in changes in adult population levels during the cold storage period were likely due to colony activity: activity in cold storage was largely limited to bee movement in and around the cluster, while colonies outside would have had warm daily temperatures and would have lost many bees in foraging efforts^25^ to support their large populations although even in a high rainfall year little was available at that time.

There is room for guarded optimism concerning further exploration of this approach. Limited cold storage itself would likely have a small or negligible impact on most colony- and individual measures of health, so the strategy may yet be a useful mite control once parameters are optimized. Temperature data indicated that brood had largely emerged after about two weeks, so time in cold storage can probably be shorter than was used here, which may reduce stress on colonies. These results suggest well nourished, as colonies were in September 2021, is not sufficient, and that levels of stressors, such as Varroa, may also need to be low. Further work is planned on the duration and timing of the cold storage.

## Materials and methods

### Field experiment 2020–21

In August 2020 fifty honey bee colonies installed in painted, 10-frame, wooden Langstroth boxes (43.7 l capacity), with marked European queens (Olivarez Honey Bees, Inc. Orland, CA) and at least 2 frames of sealed brood, were each given a second box as a super. The hives were placed on electronic scales (Tekfa model B-2418 and Avery Weigh-Tronix model BSAO1824-200) (max. capacity: 100 kg, precision: ± 20 g; operating temperature: − 30 to 70 °C) and linked to 16-bit dataloggers (Hobo UX120-006M External Channel datalogger, Onset Computer Corporation, Bourne, MA) with weight recorded every 5 min. The system had an overall precision of approximately ± 20 g. Hives were arranged in groups of 6 facing south near a central box containing electronic equipment. Hives within such a group were 0.5–1 m apart and groups were > 3 m apart. Temperature sensors (iButton Thermochron, resolution ± 0.06 °C, accuracy ± 0.5 °C, accessed using 1-Wire Drivers × 64, version 4.05) enclosed in plastic cassettes (Thermo Fisher Scientific, Waltham, MA) were stapled to the center of the top bar on the middle frame in the bottom box and set to record every 30 min and were not moved during the experiment.

Colonies were treated with amitraz (Apivar, Arysta LifeScience America Inc., New York, NY) on 1 September. Colonies were assessed on 16 September, 28 October, and finally on 10 February 2021 using a published protocol^26^. Briefly, the hive was opened after the application of smoke, and each frame was lifted out sequentially, gently shaken to dislodge adult bees, photographed using a 16.3 megapixel digital camera (Canon Rebel SL1, Canon USA, Inc., Melville, NY), weighed on a portable scale (model EC15, OHaus Corp., Parsippany, NJ), and replaced in the hive. Frame photographs were analyzed later in the laboratory (see below). During the first assessment all hive components (i.e. lid, inner cover, box, bottom board, frames, entrance reducer, internal feeder) were also shaken free of bees and weighed to yield an initial mass of all hive components.

After the first assessment, hives were ranked with respect to adult bee mass and then assigned to one of two groups: to be placed in cold storage, or kept in ambient conditions, while ensuring that the mean colony bee masses per group were approximately equal. On 24 September about 200 adult worker bees were sampled from within the brood nest of each hive and stored on ice for varroa mite screening. Each sample was washed with 70% ethanol and the number of bees and Varroa mites counted to estimate Varroa mite infestation levels, in mites per 100 bees, for each hive^27^. A second sample of about 100 bees was stored on dry ice for molecular and physiological analyses at a later date. On 30 September, and again on 20 November, all colonies were provided with 2 frames of capped honey (about 5–6 kg on average) to ensure they had enough food resources for the winter.

On 1 October 25 colonies were placed in a cold storage unit (30 m^3^ internal volume, with CO_2_ and temperature monitors, PolarKing, Fort Wayne, IN) set to 5 °C with a dehumidifier and a roof-mounted exhaust fan operating 4 min per hour. Those colonies remained in the unit until 22 October and were not opened during that period. The remaining colonies were kept outside in the original apiary. On 23 October adult bee samples were collected for physiological and molecular analyses and on 28 October a second hive assessment was conducted. Some hives were reduced to single boxes at this point. On 31 October half of the colonies that had been placed in cold storage, and half of the colonies kept in situ outside, were randomly selected and treated with thymol (Apiguard, Vita Bee Health, Basingstoke, UK), with a second dose for those colonies on 13 November. On 6 and 20 November, 2 and 18 December, and finally on 5 January 2021 all colonies were fed 200 g pollen patty, made at a ratio of 1: 1: 1 corbicular pollen (Great Lakes Bee Co.): granulated sugar: drivert sugar (Domino Foods, Yonkers, NY). Brood nest bee samples were collected and a final hive assessment was conducted on 10 February 2021.

### Field experiment 2021–22

The experiment was repeated the following year, with different colonies, following largely the same schedule. Rainfall was considerably higher prior to the experiment, leading to abundant bee forage and to considerably larger colonies. In August 2021 forty colonies were placed on scales and temperature sensors installed as described above. The temperature sensors were programmed for 15 min intervals rather than 30 min intervals. In mid-August colonies were fed with pollen patty and treated with tau fluvalinate (Apistan, Vita Bee Health, Basingstoke, UK). On 22 September any colonies that had been large enough for three boxes were reduced to two boxes. On 27 September hive evaluations were conducted and brood nest bees sampled as in the previous year, and colonies were divided into treatment groups. On 4 October half the colonies were moved to cold storage, while half the colonies remained outside in the original apiary. Colonies were again removed from cold storage after about 3 weeks. Brood nest bees were sampled, including for Varroa infestation level, and colony assessments were conducted 26–27 October. Thymol treatment was applied on 28 October and 10 November. Frames of capped honey were added to colonies on 1 and 13 December to ensure colonies had sufficient resources for the winter. Colonies were fed pollen patty on 26 October, 8 November, 1 and 8 December, 26 January, and 8 February. Final bee sampling and hive evaluations were conducted on 14 February 2022.

### Bee physiology assays

Adult bees sampled from the brood nest were immediately placed on dry ice and then stored at – 80 °C until they were processed. Material from each hive was pooled to obtain estimates of stress and gene expression levels for each hive pre- and post-storage. Adult bees were sampled before and after cold storage but before application of the miticide.

#### Oxidative damage

Oxidative damage to lipids and proteins was assayed as a proxy for overall oxidative damage in honey bees exposed to cold storage and outdoor conditions. Fifteen honey bee worker heads per colony were ground in 1 ml of ice cold phosphate buffered saline (PBS) for 1 min using a Mini-BeadBeater 96 (BioSpec Products, Oklahoma, USA). The samples were returned to ice briefly to cool, and then centrifuged for 10 min at 4 °C at 14,000 rpm (16,873×*g*). At least 500 µl of the supernatant was sampled and placed in a new tube on ice. 300 µl of this supernatant was used to measure total protein (100 µl) and oxidative damage (100 µl each to measure lipid peroxidation and protein oxidation). Total protein was assayed using a Pierce™ bicinchoninic acid (BCA) protein assay (Thermo Fisher Scientific, Massachusetts, USA). Lipid peroxidation, in the form of malonaldehyde (MDA) levels^15,28^ was measured using the OXITek™ thiobarbituric acid reactive substance (TBARS) assay according to the manufacturer’s protocol (ZeptoMetrix, New York, USA). Protein oxidation^28,29^ was measured using a Protein Carbonyl Content Assay according to the manufacturer’s protocol (Sigma-Aldrich, Missouri, USA). Minor modifications were made to the protein carbonyl assay: nucleic acids were removed prior to testing with 10 µl of streptozocin, 80 µl of the carbonyl reaction was analyzed with a spectrophotometer (Biotek Synergy HT, BioTek Instruments Inc., Vermont, USA), and 20 µl of the remaining reactant was used to measure total protein as described above and according to the Protein Carbonyl Content Assay protocol (Sigma-Aldrich, Missouri, USA)^15^. The resulting lipid and protein oxidation data were normalized to the total protein content of the supernatant.

#### Gene expression and deformed wing virus quantification

Pools of 30 bee abdomens per colony were homogenized in 2 ml of Maxwell^®^ simplyRNA homogenization solution (Promega, Madison, WI, USA) using a Bead Rupture Elite bead mill (OMNI International, Kennesaw, GA, USA). Samples were centrifuged and 200 μl of supernatant was removed for RNA extraction with a Monarch^®^ total RNA miniprep kit (New England BioLabs, Ipswich, MA, USA) according to the manufacturer’s protocol. cDNA synthesis was carried out using 1 μg of DNAase-treated RNA and a LunaScript® RT SuperMix Kit (New England BioLabs) according to the manufacturer’s protocol. Quantitative PCR (qPCR) was performed in triplicate to quantify expression levels of *vitellogenin (vg), vg-like-A, catalase, superoxide dismutase, heat shock protein 70,* and *heat shock protein 90*^23^. Viral quantification of deformed wing virus (DWV) variants DWV-A and DWV-B was performed by absolute quantification using the standard curve method and well-established protocols^30,31^.

All qPCR reactions were performed as follows: initial denaturation at 95 °C for 5 min; 40 cycles with denaturation at 95 °C for 15 s; and a primer-pair-specific annealing and extension temperature (Table 5) for 30 s. The reactions were carried out using SsoAdvanced™ Universal SYBR^®^ Green Supermix (Bio-rad, Hercules, CA, USA) in triplicate on a CFX96 Real-Time PCR Detection System (Bio-rad, Hercules, CA, USA). To confirm the absence of contaminating genomic DNA and primer dimers, negative control reactions containing only DNase-treated total RNA were tested for amplification^32^. Relative expression levels were calculated based on standardized Ct values (Δ Ct) using honey bee *β-actin* for normalization.

**Table 5:** Primers used for gene expression analyses. “Annealing temp.” is the annealing temperature.

Gene (accession number)	Forward 5′–3′	Reverse 5′–3′	Annealing temp. (°C)	References
*Actin *(XM_623378)	TGCCAACACTGTCCTTTCTG	AGAATTGACCCACCAATCCA	55.0	^33^
*vitellogenin (vg) *(AJ517411)	GTTGGAGAGCAACATGCAGA	TCGATCCATTCCTTGATGGT	57.5	^34^
*vg-like-A* XM_001121939.3	GTTTATGACGAAAATGGACACCT	TGAACAGTTTCCTCGTGAGTT	57.5	^34^
*Catalase *(NM_001178069)	TTCTACTGTGGGTGGCGAAAG	GTGTGTTGTTACCGACCAAATCC	60.0	^35^
*Sup. dismut *(NM_001178027)	TCAACTTCAAGGACCACATAGTG	ATAACACCACAAGCAAGACGAG	60.0	^35^
*HSP70 *(GB19503)	GACGCGGGAGCGATAGCAGG	AAGCCATAAGCAATCGCCGCC	60.0	^17^
*HSP90 *(GB14758)	ATGCCGGAGGACGTCACCAT	TTGTGCAATTTCAGCTTGGAAAGCG	56.0	^17^
*DWV-A *(AY292384.1)	GAG ATT GAA GCG CAT GAA CA	TGA ATT CAG TGT CGC CCA TA	54.0	^36^
*DWV-B *(AY251269)	CTG TAG TTA AGC GGT TAT TAG AA	GGT GCT TCT GGA A**T**A GCG GAA	55.0	^37^

### Data analysis

The area of sealed brood per frame was measured from photographs using ImageJ version 1.47 software (W. Rasband, National Institutes of Health, USA) or CombCount^38^, and frame values were summed to provide colony level estimates. Temperature data were transformed into daily average and within-day detrended data, calculated as the difference between the 24 h running average and the raw data. Sine curves were fit to 3-day subsamples of detrended data in C++ (Qt Creator 4.1.0) to obtain temperature amplitudes, which is a measure of temperature variability within the hive^19^. The average daily values and the amplitudes of each 3-day subsample were used as response variables. Hive weight change per day was calculated as the difference between the hive weight for a given day and the weight the previous day. To detect abrupt hive temperature changes associated with the end of sealed brood emergence in cold storage, within-hive temperature data from colonies placed in cold storage were modeled using the segmented() function in R (R Development Core Team 2020) to fit a piecewise regression line with one breakpoint. These regressions yielded estimates for 2 parameters: the break point and the adjusted r^2^.

All response variables were examined for normality using Proc Univariate (SAS, Inc.), subjected to transformation (log or arcsine of the square root) to improve normality prior to analysis if warranted, and analyzed with non-parametric rank sum tests if normality could not be obtained. Adult bee mass and brood area were subjected to two separate analyses: (1) a mixed model ANOVA using the October data with ± cold storage, year, and their interaction as fixed effects; and (2) a repeated-measures MANOVA with treatment group ( ± cold storage and  ± miticide), sampling occasion (October or February), year, and their interactions as fixed effects and an autoregressive covariance structure, ar(1) (Proc Glimmix, SAS version 9.4, SAS Inc. 2002). Pre-treatment (September) values were used as covariates in both analyses to control for pre-existing differences.

Hive weight changes and temperature averages and amplitudes after cold storage were subjected to repeated-measures MANOVA with treatment ( ± cold storage and  ± miticide), Julian day, year, and their interactions as fixed effects and pre-treatment values as covariates. Effect sizes for all significant individual and colony-level effects were calculated using Hodge’s g^39^. Colony survivorship, starting on the first day of the cold storage period, was analyzed using the mixed-model Cox regression model coxme() in R, with treatment group, year and their interaction as fixed effects.

Individual level responses (stress markers, and gene expression related to vitellogenin, antioxidant enzymes, thermal stress responses and DWV) were measured pre- and post- the cold storage period. Post-hoc comparisons with Bonferroni correction were reported for significant main effects. Stress marker data (protein carbonyl and MDA) and gene expression data were subjected to ANOVA. Cold storage, year of experiment, and their interaction were fixed effects, and September values were included in the models as covariate (Proc Glimmix, SAS version 9.4, SAS Inc. 2002).

## Data Availability

The datasets generated during and/or analysed during the current study are available from the corresponding author on reasonable request.
